# Multiple fertility restorer loci for cytoplasmic male sterility caused by mitochondrial gene *orf137* in tomato

**DOI:** 10.1093/jxb/eraf309

**Published:** 2025-07-08

**Authors:** Yurie Iki, Issei Harada, Kentaro Ezura, Seira Mashita, Kosuke Kuwabara, Hitomi Takei, Atsushi Toyoda, Kenta Shirasawa, Tohru Ariizumi

**Affiliations:** Graduate School of Life and Environmental Sciences, University of Tsukuba, Tsukuba, Ibaraki 305-8572, Japan; Graduate School of Life and Environmental Sciences, University of Tsukuba, Tsukuba, Ibaraki 305-8572, Japan; Institute of Life and Environmental Sciences, University of Tsukuba, Tsukuba, Ibaraki 305-8572, Japan; Graduate School of Life and Environmental Sciences, University of Tsukuba, Tsukuba, Ibaraki 305-8572, Japan; Graduate School of Life and Environmental Sciences, University of Tsukuba, Tsukuba, Ibaraki 305-8572, Japan; Graduate School of Life and Environmental Sciences, University of Tsukuba, Tsukuba, Ibaraki 305-8572, Japan; Advanced Genomics Center, National Institute of Genetics, Mishima, Shizuoka 411-0801, Japan; Department of Frontier Research and Development, Kazusa DNA Research Institute, Kisarazu, Chiba 292-0818, Japan; Institute of Life and Environmental Sciences, University of Tsukuba, Tsukuba, Ibaraki 305-8572, Japan; Tsukuba Plant Innovation Research Center, University of Tsukuba, Tsukuba, Ibaraki 305-8572, Japan; INRAE-Bordeaux, France

**Keywords:** Cytoplasmic male sterility, F_1_ seed production, fertility restoration, genome, tomato

## Abstract

Cytoplasmic male sterility (CMS) in plants is caused by incompatibility between nuclear and cytoplasmic genetic information. Fertility can be restored through the action of fertility restorer (*RF*) genes, which are usually present in the nucleus. CMS lines of tomato (*Solanum lycopersicum*) caused by mitochondrial gene *orf137* have been developed from asymmetric cell fusions, and in these lines, cultivated tomato served as a nuclear donor and its wild relative, *S. acaule*, as a cytoplasm donor. Although *RF* genes are present in wild relatives of tomato, no genetic or genomic information on the *RF* genes is yet available. This study reports an *RF* genetic locus, *RF1*, on chromosome 1 of *S. pimpinellifolium* LA1670 and *S.*  *lycopersicum* var. *cerasiforme* LA1673, which was revealed by bulked segregant analysis and sequencing. An additional *RF* locus, *RF2*, was identified on chromosome 2 of LA1670. The genomic sequence of *S. cheesmaniae* LA0166 was assembled using high-fidelity, long-read sequencing technology. Sequence comparisons identified further candidate *RF* genes on chromosome 1 of *S. cheesmaniae* LA0166. These results suggested that multiple gene loci control the fertility restoration trait in wild relatives of tomato.

## Teaser

Tomato cytoplasmic male sterility caused by incompatibility between nuclear and cytoplasmic genetic information is restored through the action of multiple fertility restorer genes of tomato wild relatives.

## Introduction

Cultivated and wild species have evolved separately, and so even when two species are cross-compatible, their genetic information may be incompatible between species (Orr, 1996). Following the generation of an interspecific hybrid, recurrent backcrossing, intended to replace a wild nuclear gene with a cultivated one, or vice versa, can lead to male sterility due to incompatible interactions between the nuclear and mitochondrial genomes. This is known as cytoplasmic male sterility (CMS). CMS occurs when plants lack nuclear genes able to regulate mitochondrial gene expression, thus exposing them to the deleterious effects of mitochondrial genes (Toriyama *et al.*, 2024). Such a mitochondrial gene is often called a CMS-associated gene, and its product affects mitochondrial function and the regulation of nuclear genes, leading to male sterility (Toriyama, 2021). As fertility can be restored by nuclear genes that are compatible with the mitochondrial genes, the gene regulators encoded in the nuclear genome are called fertility restorer (*RF*) genes.

It is known that there are two modes of fertility restoration in CMS, sporophytic and gametophytic modes (Toriyama, 2021). In the sporophytic type, the pollen fertility of the F_1_ plant (*RFrf*) between a CMS line (*rfrf*) and a restorer line (*RFRF*) is determined by the genotype of the sporophyte (*RFrf*), resulting in all the pollen (*RF* and *rf*) being fertile. In the gametophytic type, on the other hand, the genotype of individual pollen grains (*RF* and *rf*) determines their fertility, in which *rf* pollen is aborted. Therefore, in the F_2_ generation, both fertile (*RFRF* and *RFrf*) and sterile (*rfrf*) plants are expected in sporophytic-type CMS, while only fertile plants (*RFRF* and *RFrf*) are expected in the gametophytic type.

CMS lines of tomato (*Solanum lycopersicum*), which is one of the most important food crops worldwide, were developed by asymmetric cell fusions that used cultivated tomato as a nuclear donor and its wild relative, *S. acaule*, as a cytoplasm donor (Melchers *et al.*, 1992). A mitochondrial gene, *orf137*, triggers CMS in the fusion lines (Kuwabara *et al.*, 2022). *Solanum pimpinellifolium* LA1670, *S. lycopersicum* var. *cerasiforme* LA1673, and *S. cheesmaniae* LA0166, three wild relatives of tomato, possess *RF* genes that restore fertility to the CMS lines (Harada *et al.*, 2003); to date, however, no *RF* genetic loci have been reported in tomato.

CMS is a useful trait for F_1_ seed production because emasculation, which is labor-intensive and thus costly, is not required for outcrossing. In crops whose product is set fruit and seeds, however, the paternal lines of F_1_ hybrids must possess *RF* genes to ensure fertility in their F_1_ progeny, whose cytoplasm contains CMS genes. Identification of *RF* gene loci is thus an important aspect of efficient breeding in the paternal lines. Pentatricopeptide repeat (PPR) proteins act as *RF* genes in many crop plants (Toriyama, 2021). These genes are often tandemly arrayed in *RF* regions of genomes (Geddy and Brown, 2007); it is also known that the number of copies of *PPR* genes varies between *RF* loci and that this copy number variation functions in fertility restoration (Wang *et al.*, 2006).

As the genomes of non-RF tomato lines may lack functional *RF* gene sequences, the fully sequenced genomes from RF lines are useful tools in the identification of *RF* genes. The genomic sequences of *S. pimpinellifolium* LA1670 and *S. lycopersicum* var. *cerasiforme* LA1673, two of the three relatives of tomato that possess *RF* genes, are publicly available (Takei *et al.*, 2021), although the genome of the third wild relative, *S. cheesmaniae* LA0166, is not. In this study, we used a map-based cloning strategy with the genomic sequences of the RF lines to identify genetic loci for *RF* genes in all three tomato relatives. Furthermore, we determined the genomic sequence of *S. cheesmaniae* LA0166. Finding the locations of these *RF* genes, together with the development of linked DNA markers, is a major step towards the identification of *RF* genes in tomato, which will enable the efficient breeding of elite RF paternal lines for use in tomato F_1_ seed production. Furthermore, genetic and genomic dissection of the *RF* genes from the three species would provide insights into the evolutional history and molecular mechanisms of the RF system in tomato.

## Materials and methods

### Plant materials

**Fig. 1.**
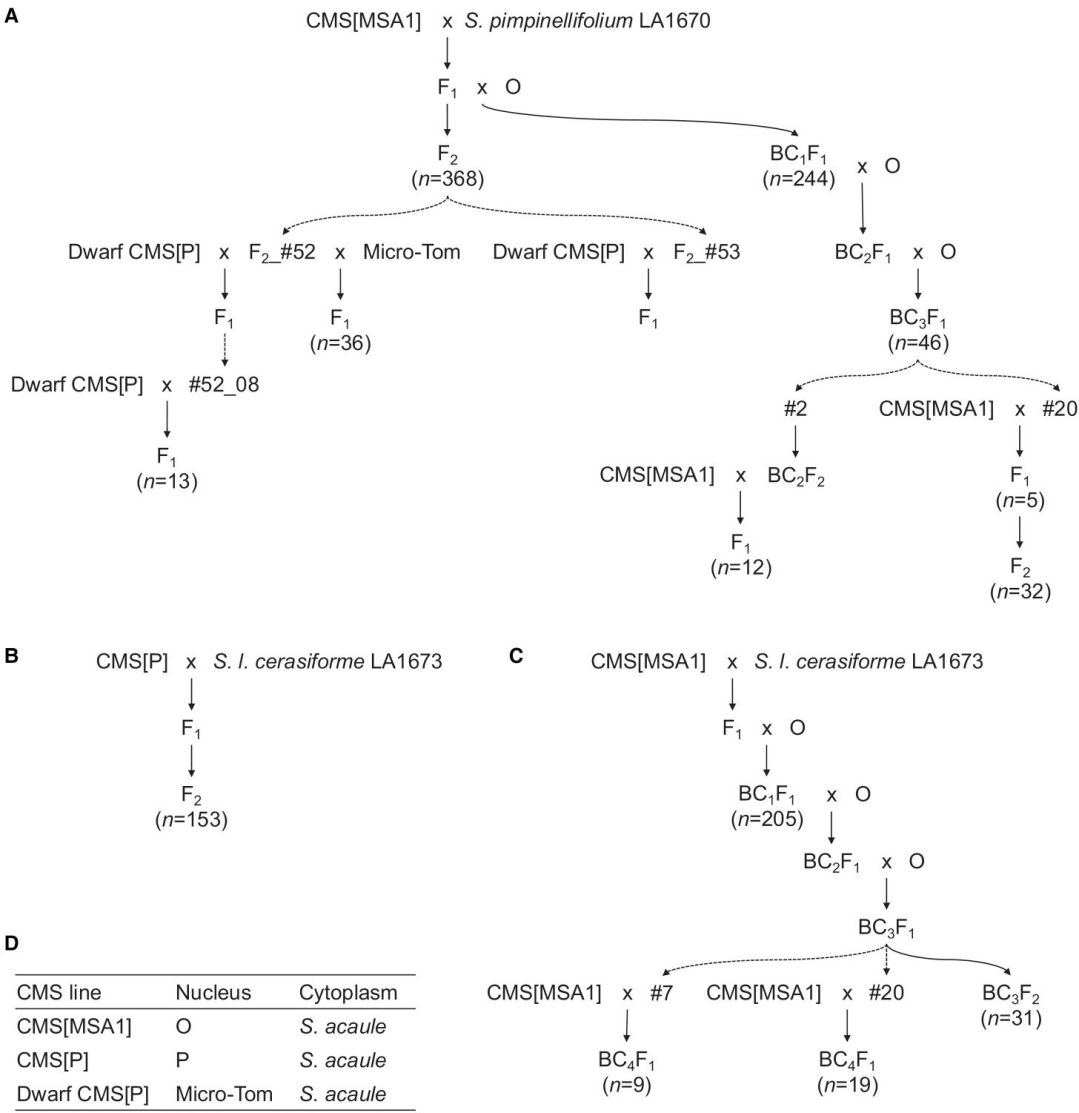
Pedigrees of plant materials. (A) The pedigree of *Solanum. pimpinellifolium* LA1670 to identify *RF1a1* and *RF2*. (B, C) The pedigree of *Solanum lycopersicum* var. *cerasiforme* LA1673 to identify *RF1a2*. Arrows and arrows with dashed lines indicate self-pollinated or crossed progeny and individual selection, respectively. Line names on the left and right sides of crosses indicate the female and male parent, respectively, i.e. female×male. (D) Genomes of nucleus and cytoplasm; the pedigrees are described in our previous publication (Kuwabara *et al.*, 2021).

The full details of the pedigrees of the tomato lines used in this study are shown in Fig. 1. The tomato (*Solanum lysopersicum*) cultivars O and Micro-Tom, both of which lack CMS genes in the cytoplasm and *RF* genes in the nucleus, were used in experiments. We also used the CMS tomato lines CMS[MSA1] and CMS[P], which were described in our previous study (Kuwabara *et al.*, 2021). CMS[MSA1] was developed by repeated backcrossing using O as a paternal recurrent parent and the maternal male-sterile line MSA1 as a cytoplasmic donor; MSA1 is an asymmetric cell fusion line derived from the cultivar Sekai-ichi (nuclear donor) and a wild relative, *S. acaule* (cytoplasmic donor) (Melchers *et al.*, 1992). CMS[P] was produced by backcrosses in which the cultivar P (Kuwabara *et al.*, 2021) served as the paternal recurrent parent and an asymmetric cell fusion between P (nuclear donor) and *S. acaule* (cytoplasmic donor) as the maternal line (Kuwabara *et al.*, 2021). Both CMS lines carry the CMS gene, *orf137*, in their mitochondria (Kuwabara *et al.*, 2022). In addition, we used Dwarf CMS[P], a line developed by repeatedly backcrossing CMS[P] with Micro-Tom [TOMJPF0001 provided by University of Tsukuba, Tsukuba Plant Innovation Research Center, through the National Bio-Resource Project (NBRP) of the MEXT/AMED, Japan] (Kuwabara *et al.*, 2021). This Dwarf CMS[P] produces non-germinating pollens (Kuwabara *et al.*, 2022).

Three wild relatives of tomato, *S. pimpinellifolium* LA1670, *S. lycopersicum* var. *cerasiforme* LA1673, and *S. cheesmaniae* LA0166, were used as RF lines. Seeds from these species were obtained from the Tomato Genetic Resource Center at the University of California, Davis, USA.

All plants including fertile and sterile lines were simultaneously grown in a greenhouse at the University of Tsukuba under natural light conditions in the spring and autumn season for phenotype evaluation.

### Pollen germination test

Phenotyping of pollen tube growth was performed using the method described in Kuwabara *et al*. (2022). For *in vivo* pollen germination tests on stigmas, pistils were fixed in ethanol: acetic acid (3:1, v/v) 24 h after self-pollination. Fixed pistils were soaked in 5 M NaOH for 24 h. After washing three times, pistils were stained in the dark for 24 h with 0.001 w/v% aniline blue in 0.1 M K_2_HPO_4_ buffer (pH 10).

Pollen germination tests using liquid germination medium were performed according to a previously described protocol with minor modifications (Steven and de Wouter, 1993). Freshly opened flowers were soaked in 1 ml germination medium [15.1 w/v% polyethylene glycol (average molecular weight: 6000), 10 w/v% sucrose, 1.63 mM H_3_BO_3_, 1.27 mM Ca(NO_3_)_2_, 1 mM MgSO_4_, 1 mM KNO_3_, and 0.1 mM K_2_HPO_4_]. The mixture was strongly vortexed to release the pollen from the anthers. The flower residues were removed and the pollen suspension was incubated in the germination medium and agitated using a rotator at 25 °C for 4 h. Pollen tubes were observed using a BX53 microscope (Olympus, Hachioji, Japan). Digital images were captured using a DP72 camera (Olympus).

### DNA extraction and PCR analysis

Genomic DNA was isolated from young leaves using a DNeasy Plant Mini Kit (Qiagen, Hilden, Germany) and a Maxwell 16 Purification Kit (Promega, Madison, WI, USA). Each 10 µl PCR contained 0.5 µl genomic DNA, 0.3 µM primers (Supplementary Table S1), 2× PCR buffer (Toyobo, Osaka, Japan), 400 µM dNTPs, and 1 U DNA polymerase (KOD FX Neo, Toyobo). The thermal cycling conditions were as follows: initial denaturation at 94 °C for 3 min; 35 cycles of denaturation at 98 °C for 15 s, annealing at 55–60 °C for 30 s, and extension at 68 °C for 60 s; followed by a final extension at 68 °C for 3 min. The PCR products were digested with restriction enzymes and separated by electrophoresis through 1% agarose gels submerged in Tris-acetate–EDTA (TAE) buffer. Gels were stained with Midori Green Advance (Nippon Genetics, Tokyo, Japan) to visualize DNA bands under ultraviolet illumination (312 nm) using a UV transilluminator (E-BOX VX2/20M, Vilber Lourmat, France).

### Bulked segregant analysis with short-read sequencing

A whole genome shotgun library was prepared using TruSeq DNA PCR-Free Sample Prep Kit (Illumina, San Diego, CA, USA). A double-digest restriction site-associated DNA sequencing (ddRAD-Seq) library was constructed as described previously (Shirasawa *et al.*, 2016) with minor modifications. Genomic DNA was digested with the restriction enzymes *Pst*I and *Msp*I (Fast Digest restriction enzymes; Thermo Fisher Scientific, Waltham, MA, USA). Nucleotide sequences were determined using short-read DNA sequencers manufactured by Illumina and MGI Tech (Shenzhen, China). Data processing and single nucleotide polymorphism (SNP) identification were performed as described previously (Shirasawa *et al.*, 2016). High-quality sequence reads were mapped onto the reference sequence to identify high-confidence SNPs. SNPs associated with the traits were identified and visualized using QTLseqr (Mansfeld and Grumet, 2018).

### Genome assembly and gene annotation of LA0166

LA0166 genome assembly was performed as described previously (Shirasawa and Ariizumi, 2024). High molecular weight genomic DNA was isolated from leaves of LA0166 using Genomic-Tips (Qiagen). gDNA libraries were constructed using the SMRTbell Express Template Prep Kit 2.0 (PacBio, Menlo Park, CA, USA). The resulting libraries were fractionated with BluePippin (Sage Science, Beverly, MA, USA) to eliminate fragments shorter than 20 kb and sequenced on a SMRT Cell 8M using the Sequel II system (PacBio). HiFi reads were constructed using the CCS pipeline (https://ccs.how) and assembled using Hifiasm with default parameters. The assembly was aligned on the SL4.0 genomic sequence (Tomato Genome Consortium, 2012) to establish a chromosome-level sequence with RaGoo (Alonge *et al.*, 2019).

Gene prediction was performed using BRAKER3 (Gabriel *et al.*, 2024), based on the peptide sequences of the genes predicted by ITAG4.0 (https://solgenomics.net) and Iso-Seq reads obtained from anthers of LA0166, according to the PacBio standard protocol (PacBio). The gene sequences reported by ITAG4.0 were then mapped onto the pseudomolecule sequences using Liftoff (Shumate and Salzberg, 2021). The completeness of the genome assembly and gene prediction were evaluated with embryophyta_odb10 data using benchmarking universal single-copy orthologs (BUSCO) (Simão *et al.*, 2015). Repetitive sequences in the assembly were predicted by RepeatMasker (https://www.repeatmasker.org) using repeat sequences registered in Repbase, and a *de novo* repeat library was built using RepeatModeler (https://www.repeatmasker.org). Genome structures were compared with MCScanX (Wang *et al.*, 2012) and visualized using SynVisio (Bandi and Gutwin, 2020).

## Results

### *RF1a1* in *S. pimpinellifolium* LA1670

The mode of fertility restoration in *S. pimpinellifolium* LA1670 was investigated using an F_2_ population (*n*=368) derived from the cross CMS[MSA1]×LA1670 (Fig. 1A). All but five F_2_ plants achieved seed set by self-pollination. Pollen from the five plants that did not set fruit, or set seedless fruits due to parthenocarpy, was able to germinate. The result indicated that all F_2_ plants tested were fertile, suggesting that the gametophytic mode of pollen fertility restoration was present in LA1670.

Subsequently, a BC_1_F_1_ population (*n*=244) was generated by crossing an F_1_ hybrid (derived from CMS[MSA1]×LA1670) with O (Fig. 1A). When the BC_1_F_1_ population had grown to maturity, 133 plants showed seed formation and/or pollen germination, whereas the remaining 113 plants exhibited no pollen germination, indicating male sterility. The segregation ratio of fertile to sterile plants was close to 1:1 (χ^2^=1.328, *P*=0.249), suggesting that fertility restoration was controlled by a single genetic locus.

**Fig. 2.**
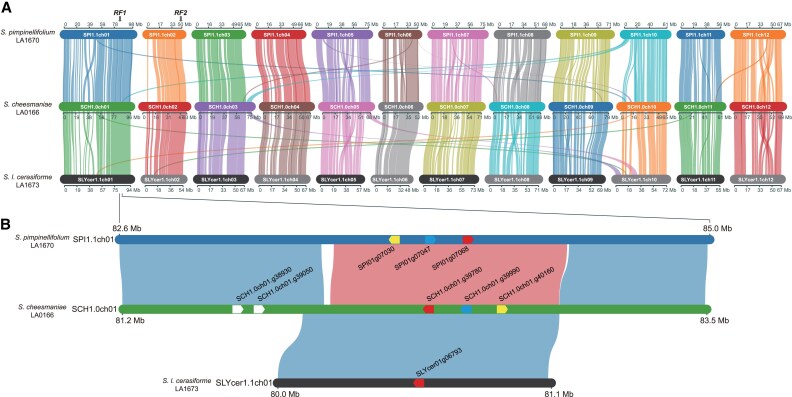
Genomic structures of the LA1670, LA0166, and LA1673 RF lines. (A) Chromosome structural similarity between the three RF lines of LA1670 (SPI1.1ch01 to SPI1.1ch12), LA0166 (SCH1.0ch01 to SCH1.0ch12), and LA1673 (SLYcer1.1ch01 to SLYcer1.1ch12). Arrows indicate the *RF1* and *RF2* loci. (B) Genomic structural similarity between the *RF1* loci on chromosome 1 of the three RF lines. Blue and red alignment links between the RF lines show regular and reversed orientations, respectively. Pentagons indicate *PPR* genes with gene ID colors (yellow, blue, and red) indicating orthologous genes.

To identify the genetic locus underlying fertility restoration, we performed bulked segregant analysis by sequencing. Sequence reads, obtained from bulked samples of the fertile and sterile BC_1_F_1_ plants, were mapped onto the genomic sequence of *S. pimpinellifolium* LA1670. This analysis suggested that an *RF* candidate gene was located in a 10 Mb region situated between approximately 80 and 90 Mb of chromosome 1 (Supplementary Fig. S1). Next, 46 BC_3_F_1_ plants, generated by backcrosses between a fertile BC_1_F_1_ plant as a donor and O as a recurrent parent (Fig. 1A), were genotyped using 31 cleaved amplified polymorphic sequence (CAPS) markers designed against the candidate region. This analysis narrowed the candidate region down to a 2.4 Mb region between 82.6 and 85.0 Mb of chromosome 1 (Fig. 2). This region was named *RF1a1*.

In this process, we found that the fertility restoration in this tomato CMS system is gradual, not binary (Fig. 2; Supplementary Table S2). We selected two individuals from the 46 BC_3_F_1_ plants, #2 and #20, both of which were male fertile. One plant (#2) was self-pollinated to obtain a male-fertile BC_2_F_2_ plant (#2-17), which set a mean 3.75 seeds per fruit. This BC_2_F_2_ plant (#2-17) was crossed with CMS[MSA1] to obtain 12 F_1_ hybrids. While three plants did not set seeds probably due to male-sterility, nine plants set seeds ranging from 0.3 to 14.7 seeds/fruit. The other plant (#20) was also crossed with CMS[MSA1] to obtain five male-fertile F_1_ plants (#20-2, -4, -9, -13, and -18) setting 0.1–6.3 seeds/fruit. These self-pollinated seeds from the five plants were sown to obtain a total of 32 F_2_ plants. In this case, while seven plants did not set seeds, the remaining 25 plants set 0.1–20.3 seeds/fruit.

### *RF2* in *S. pimpinellifolium* LA1670

In the F_2_ population (*n*=368) from the cross CMS [MSA1]×LA1670 (Fig. 1), 366 fertile plants carried the functional *RF1* allele. However, two plants (F_2__#52 and F_2__#53) exhibited male fertility despite being homozygous for the non-functional *rf1* allele, suggesting the presence of an alternative restoration mechanism. To confirm the inheritance pattern of the fertility restoration, the two fertile F_2_ plants were crossed with Dwarf CMS[P] (Fig. 1A). These crosses produced two fertile F_1_ plants (#52_08 and #53_08), neither of which contained the LA1670 allele of *RF1*. This finding indicated the presence of another novel *RF* locus that was distinct from *RF1*.

To identify this novel *RF* locus, henceforth called *RF2*, plant #52_08 was crossed with Dwarf CMS[P]; this cross produced 13 F_1_ plants (Fig. 1A). In addition, plant F_2__#52 was crossed with Micro-Tom; this cross produced 36 F_1_ plants (Fig. 1A). As expected, all progeny from the cross Dwarf CMS[P]×F_2__#52 were fertile, but the progeny from the cross F_2__#52×Micro-Tom segregated into 28 fertile to 8 sterile plants, fitting the Mendelian segregation ratio of a single gene (χ^2^=0.15, *P*=0.70). Of the 49 F_1_ plants produced by these two crosses, 13 fertile and eight sterile plants were selected for bulked segregant analysis. Sequence reads obtained from the genomic DNA of the bulked sterile and bulked fertile plants were mapped onto the genomic sequence of *S. pimpinellifolium* LA1670. This identified a 10 Mb *RF2* candidate region that lay between approximately 40.0 and 49.8 Mb of chromosome 2 (Fig. 2; Supplementary Fig. S1).

### *RF1a2* in *S. lycopersicum* var. *cerasiforme* LA1673

We tested the mode of fertility restoration in *S. lycopersicum* var. *cerasiforme* LA1673, using an F_2_ population (*n*=153) derived from the cross CMS[P]×LA1673 (Fig. 1B). All but two F_2_ plants set seeds by self-pollination. Pollen germination was observed, however, in the two plants that did not set fruit, which suggested that *S. lycopersicum* var. *cerasiforme* LA1673 also possessed the gametophytic mode of pollen fertility restoration.

A BC_1_F_1_ population (*n*=205) was generated by a cross between an F_1_ hybrid (derived from the cross CMS[MSA1]×LA1673) and O (Fig. 1C). When the BC_1_F_1_ plants had grown to maturity, we observed seed formation and/or pollen germination in 109 plants, but neither fruit setting nor pollen germination was exhibited by the remaining 96 plants, indicating male sterility. The segregation ratio of fertile to sterile plants was close to 1:1 (χ^2^=0.824, *P*=0.364), indicating that a single gene controlled fertility restoration in this population.

To identify the locus underlying fertility restoration, 106 fertile and 77 sterile BC_1_F_1_ plants were subjected to ddRAD-Seq analysis. The obtained reads from fertile and sterile plants were separately merged to generate bulked data and mapped onto the LA1673 genome sequence. This analysis identified an *RF* locus located in an 80 Mb region of chromosome 1 (Supplementary Fig. S1). To identify the *RF* locus more precisely, fertile BC_1_F_1_ plants (female parent) were back-crossed twice with O (male parent) to generate a BC_3_F_1_ population (Fig. 1C). A single fertile BC_3_F_1_ plant was self-pollinated to generate a BC_3_F_2_ population (*n*=31). In addition, pollens from two fertile plants, #7 and #20, were pollinated to CMS[MSA1], producing nine and 19 BC_4_F_1_ plants, respectively. The genotypes of all 59 plants were analysed using 10 CAPS markers. Subsequently, 13 recombinant plants were analysed further using two additional CAPS markers. This analysis indicated that the *RF* locus was located in a 1.1 Mb region between 80.0 and 81.1 Mb of chromosome 1 (Fig. 2). This *RF* locus was named *RF1a2*.

### *RF1d* in *S. cheesmaniae* LA0166

We considered that obtaining the genomic sequence of LA0166 would help us identify *RF* genes in LA0166. We therefore assembled 17.5 Gb HiFi reads obtained from LA0166 into 443 contig sequences that had a total length of 835.6 Mb (Supplementary Table S3). The contigs were aligned with the tomato genome, SL4.0, to establish 12 pseudomolecules, i.e. SCH_r1.0.pmol (Supplementary Table S4). A total of 31 356 genes were predicted from the genomic sequence (Supplementary Table S4). Repetitive sequences occupied 73.4% of the genome (Supplementary Table S5). The accuracy of the genome assembly and gene predictions were supported by BUSCO scores of 98.5% and 93.8%, respectively (Supplementary Table S6). A comparative analysis of genomic structures revealed that, although the sequences of the 12 pseudo-chromosomes essentially corresponded with the chromosomes of LA1670 and LA1673 (Fig. 2A), several chromosome rearrangements were present, as we detected inversions in chromosomes 1, 5, 8, and 12 and translocations in chromosomes 1, 3, 5, 8, 9, 10, and 11.

The *RF1* locus of LA1670 corresponded with a 2.4 Mb region of LA0166 situated between 81.2 Mb and 83.5 Mb of chromosome 1 (Fig. 2); a segmental inversion was detected in the region between 83.4 Mb and 84.4 Mb of LA1670. The *RF1* locus of LA1673 also corresponded with a 1.1 Mb region situated between 81.9 Mb and 82.9 Mb of LA0166 (Fig. 2). The 2.4 Mb region of LA0166 was tentatively named the *RF1d* locus. It contained five *PPR* gene copies (*SCH1.0ch01.g38930*, *SCH1.0ch01.g39050*, *SCH1.0ch01.g39780*, *SCH1.0ch01.g39990*, and *SCH1.0ch01.g40160*), whereas the *RF1* candidate regions of LA1670 and LA1673 contained three (*SPI01g07030*, *SPI01g07047*, and *SPI01g07068*) and one (*SLYcer01g06793*) *PPR* gene copies, respectively (Fig. 2B). In accordance with the gene collinearity detected in these regions, three of the *PPR* gene copies (*SCH1.0ch01.g39780*, *SCH1.0ch01.g39990*, *SCH1.0ch01.g40160*) detected in LA0166 may be orthologs of the three copies detected in LA1670. By contrast, two of the three copies were absent from LA1673, although the remaining copy (*SLYcer01g06793*) may be an ortholog of *SCH1.0ch01.g39780*. In addition, LA0166 possessed two additional *PPR* gene copies (*SCH1.0ch01.g38930* and *SCH1.0ch01.g39050*), which were absent from the LA1670 genome (Fig. 2).

## Discussion

We identified an *RF* locus in the same region of chromosome 1 of LA1670 and LA1673, two relatives of tomato (Fig. 2). As synteny was observed in this region, this *RF* gene may be allelic or orthologous between the two species. We named this region the *RF1* locus, and therefore hypothesized that these *RF* genes might also be located in the corresponding region of chromosome 1 of LA0166 (Fig. 2). Another *RF* locus was identified in chromosome 2 of LA1670 and named the *RF2* locus (Fig. 2A). In parallel, we found the fertility restoration in this CMS tomato is a quantitative trait rather than qualitative (Supplementary Table S2). These results suggested that a major *RF* locus (*RF1*) along with minor loci (*RF2*, etc.) from tomato relatives might be involved in the RF system in tomato. These genes may be derived from natural variation. Our results suggested that pyramiding the different *RF* genes into a single plant would generate highly efficient *RF* lines. The SNPs linked to the *RF* loci identified in this study (Supplementary Table S1) will be useful DNA markers for marker-assisted selection in breeding programs to produce *RF* lines through gene pyramiding.

*PPRs* have been identified as *RF* genes in many plant species (Toriyama, 2021). In some species, *PPRs* are tandemly arrayed in the *RF* regions (Geddy and Brown, 2007). However, as other *PPR* genes were also present in the candidate regions (Fig. 2), further studies are required to determine which of these genes are responsible for *RF* function. Non-*PPR* genes can also function as *RF* genes in plants (Toriyama, 2021). In maize (*Zea mays*), a gene encoding aldehyde dehydrogenase acts as an *RF* gene that restores CMS caused by Texas (T) cytoplasm (Liu *et al.*, 2001), although the molecular mechanism remains unclear. In rice (*Oryza sativa*), a gene encoding a glycine-rich protein that degrades *atp6*-*orf79* mRNA, thus reducing ORF79 protein levels, also acts as an *RF* gene to cause Lead Rice-type CMS (Itabashi *et al.*, 2011). Another exception in rice is an *RF* gene that encodes RETROGRADE-REGULATED MALE STERILITY (Fujii and Toriyama, 2009), whose expression is up-regulated by retrograde signals from *orf307*, a mitochondrial gene that causes Chinese wild rice-type CMS. The possibility that non-*PPR RF* genes are present in tomato should therefore be kept in mind when seeking to identify *RF* genes.

In general, *RF* genes restore male fertility by suppressing products and/or signals from CMS genes (Toriyama, 2021). Further investigations are therefore required to clarify the molecular mechanisms underlying CMS and its restoration in this case. These investigations should consider multiple aspects of gene regulation, including gene copy number, mRNA processing and degradation, inhibition of translation, structural variation in proteins and protein complexes, and detoxification of CMS gene products. A multi-omics approach, including transcriptomic, proteomic, and metabolomic analyses in addition to genomics and genetics, could be used to identify *RF* genes in tomato. The current study is an important advance in the identification of tomato *RF* genes. Its results will be useful in F_1_ seed production using CMS tomato lines, which currently requires a high investment of labor and cost due to the need for emasculation and pollination by hand.

## Supplementary Material

eraf309_Supplementary_Data

## Data Availability

Raw read data were deposited in the Sequence Read Archive (SRA) database of the DNA Data Bank of Japan (DDBJ) under the BioProject accession number PRJDB19708. The assembled LA0166 sequences are available at DDBJ (accession numbers AP038850–AP038861), for which predicted gene information (coding sequences, putative peptide sequences, and their genome positions) are available at Kazusa Genome Atlas (https://genome.kazusa.or.jp).

## Supplementary data

The following supplementary data are available at *JXB*  online.

Fig. S1. Bulked segregant analysis by sequencing.

Table S1. CAPS markers linked to the *RF1a2* locus.

Table S2. Number of seeds per fruit in the progeny of a cross between CMS[MSA1] and LA1670.

Table S3. Assembly statistics of the LA0166 genome.

Table S4. Completeness of the genome assembly and gene prediction.

Table S5. Chromosome-level assembly of the LA0166 genome.

Table S6. Repetitive sequences in the LA0166 genome.

## Abbreviations

BUSCO: benchmarking universal single-copy orthologs
CAPS: cleaved amplified polymorphic sequence
CMS: cytoplasmic male sterility
RF: fertility restorer
PPR: pentatricopeptide repeat
