# Supplementary material for: Multiple fertility restorer loci for cytoplasmic male sterility caused by mitochondrial gene orf137 in tomato
Source: J Exp Bot. 2025 Jul 8;76(21):6520–6. doi: 10.1093/jxb/eraf309 (PMC12646146; doi:10.1093/jxb/eraf309)
Supplement: eraf309_Supplementary_Data [file eraf309_supplementary_data.zip › jexbot315045-file001.pdf]

Supplementary Information

Multiple fertility restorer loci for cytoplasmic male sterility caused by *orf137* in tomato

Yurie Iki[1,#], Issei Harada[1,#], Kentaro Ezura[2], Seira Mashita[1], Kosuke Kuwabara[1], Hitomi Takei[1], Atsushi Toyoda[3], Kenta Shirasawa[4,*], Tohru Ariizumi[2,5,†]

[1]Graduate School of Life and Environmental Sciences, University of Tsukuba, Tsukuba, Ibaraki 305-8572, Japan

[2]Institute of Life and Environmental Sciences, University of Tsukuba, Tsukuba, Ibaraki 305-8572, Japan

[3]Advanced Genomics Center, National Institute of Genetics, Mishima, Shizuoka 411-0801, Japan

[4]Department of Frontier Research and Development, Kazusa DNA Research Institute, Kisarazu, Chiba 292-0818, Japan

[5]Tsukuba Plant Innovation Research Center, University of Tsukuba, Tsukuba, Ibaraki 305-8572, Japan

[*]To whom correspondence should be addressed.

Tel.: +81-438-52-3935; Fax: +81-438-52-3934; Email: shirasaw@kazusa.or.jp

[#]These two authors contributed equally to this work.

Supplementary Table S1. CAPS markers linked to the *RF1a2* locus.

Supplementary Table S2. Number of seeds per fruit in the progeny of a cross between CMS[MSA1] and LA1670.

Supplementary Table S3. Assembly statistics of the LA0166 genome.

Supplementary Table S4. Completeness of the genome assembly and gene prediction.

Supplementary Table S5. Chromosome-level assembly of the LA0166 genome.

Supplementary Table S6. Repetitive sequences in the LA0166 genome.

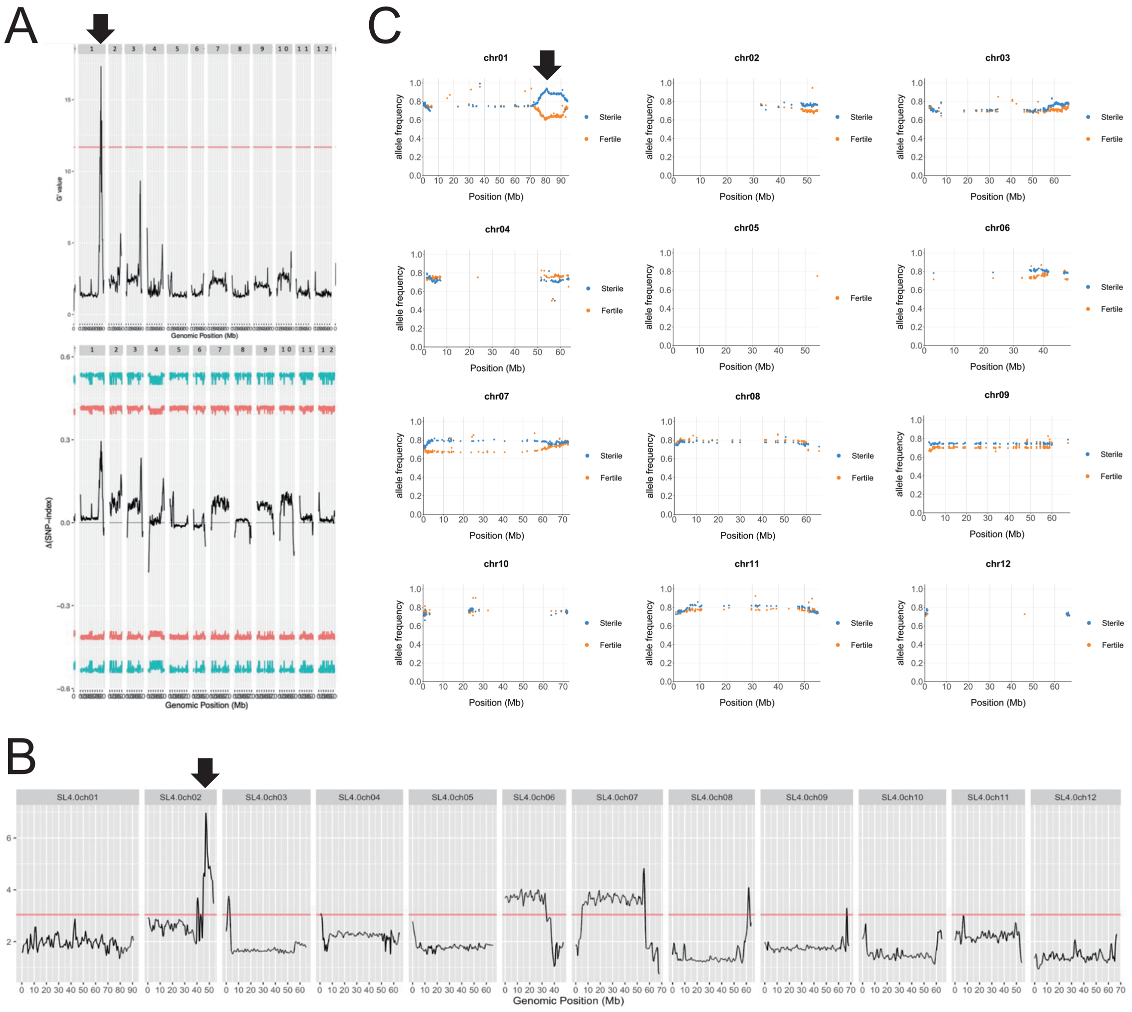

Supplementary Figure S1. Bulked segregant analysis by sequencing.

Arrows indicate the chromosomal positions of *RF1a1* (A), *RF2* (B), and *RF1a2* (C).
